# Effects of the HN Antigenic Difference between the Vaccine Strain and the Challenge Strain of Newcastle Disease Virus on Virus Shedding and Transmission

**DOI:** 10.3390/v9080225

**Published:** 2017-08-15

**Authors:** Jingjing Liu, Jie Zhu, Haixu Xu, Juan Li, Zenglei Hu, Shunlin Hu, Xiaoquan Wang, Xiufan Liu

**Affiliations:** 1 College of Veterinary Medicine, Yangzhou University, Yangzhou 225009, China; yzdxliujingjing@163.com (J.Liu); zhujievet@163.com (J.Z.); 006634@yzu.edu.cn (H.X.); li198820062011@163.com (J.Li); zengleihu@163.com (Z.H.); slhu@yzu.edu.cn (S.H.); springyz@yahoo.com.cn (X.W.); 2 Jiangsu Co-innovation Center for Prevention and Control of Important Animal Infectious Diseases and Zoonoses, Yangzhou University, Yangzhou 225009, China

**Keywords:** Newcastle disease virus, vaccination, antigenic difference

## Abstract

Newcastle disease (ND) leading to heavy economic losses to the poultry industry worldwide is caused by Newcastle disease virus (NDV). Even though intensive vaccination programs have been implemented in many countries, virulent NDV can still be frequently isolated in well-vaccinated flocks. We compared the protection efficiency of LaSota and two sub-genotype VIId vaccines, NDV/AI4 and NDV O/AI4, in which NDV O/AI4 was constructed by replacing the hemagglutinin–neuraminidase (HN) gene of the vaccine strain NDV/AI4 with that from the variant NDV strain JS-14-12-Ch by the cross hemagglutination inhibition test and immune protection test. The number of birds shedding the virus and the titer of the shedding virus from the challenged birds were tested to evaluate the protection efficiency in the immune protection test. The cross hemagglutination inhibition and neutralization tests between JS-14-12-Ch and the three vaccines displayed a significant antigenic difference between JS-14-12-Ch and LaSota or NDV/AI4, but not between JS-14-12-Ch and NDV O/AI4. The results of the immune protection test showed that NDV O/AI4 could provide improved protection as determined by a significant decrease in both the number of birds shedding the virus and the titer of the shedding virus from the challenged birds. The results in this study indicated that the antigenic similarity between the vaccine strain and the challenge strain is important in reducing the shedding of virulent virus in which the congruence of the NDV HN protein may play a critical role.

## 1. Introduction

Newcastle disease (ND), which leads to heavy economic losses to the poultry industry, is caused by virulent Newcastle disease virus (NDV) [1]. Intensive vaccination programs have been implemented in most poultry farms in China and the vaccine strain LaSota, which belongs to genotype II, has been the most extensively used since the 1970s. However, infections of genotype VII NDVs have been frequently reported in China and other Asian countries in more recent years [2,3,4,5,6,7]. In addition, the isolation rate of the sub-genotype VIId NDV strains with the E347K variation in the hemagglutinin–neuraminidase (HN) protein has increased in China over the years [6]. Moreover, researchers have reported that the HN protein would intensify the antigenic difference [8]. 

It is widely recognized that the ND vaccines which are phylogenetically closer to circulating field viruses appear to be more efficient in ND prevention in terms of reducing the virulent virus load and shedding [7,9,10]. Moreover, researchers have recently found that when the level of humoral antibody titers increased in vaccinated birds, the number of infected birds and the amount of virulent NDV shed would be decreased [11,12,13].

The objective of this study is to investigate the effects of the antigenic difference in the HN protein between the vaccine strain and the challenge strain on the protection efficiency in terms of the virulent virus load and shedding. Toward this end, using reverse genetics we generated a vaccine strain designated as NDV O/AI4 by replacing the HN gene of the vaccine strain NDV/AI4 with that of the variant NDV strain JS-14-12-Ch bearing the E347K and G362A co-mutation, while NDV/AI4 was attenuated from the NDV JS5/05 strain [14,15,16,17]. We compared the immune efficiency difference between LaSota, AI4 and NDV O/AI4 against the challenge strain JS-14-12-Ch, while the antibody titers of the vaccinated birds with each of the three vaccines were at the same level. Our results indicated that the antigenic similarity between the vaccine strain and challenge strain is important in reducing the shedding of virulent virus in which the congruence of the NDV HN protein may play a critical role.

## 2. Methods

### 2.1. Ethics Statement

Animal experiments were approved by the Jiangsu Administrative Committee for Laboratory Animals (Permission number: SYXK-SU-2007-0005), and complied with the guidelines of Jiangsu laboratory animal welfare and ethical of Jiangsu Administrative Committee of Laboratory Animals.

At the end of the animal experiments, compressed CO_2_ from gas cylinders was used for euthanasia. Lack of responsiveness to manual stimulation or immobility is defined as a clinically irreversible condition leading inevitably to death and used as an endpoint to determine when to euthanize the animals in control group.

### 2.2. Cells, Plasmids and Viruses

BHK-21 cells (clone BSR-T7/5), generated by Buchholz [18], were a gift from Zhigao Bu (Harbin Veterinary Institute, Harbin, China). Three helper plasmids (pCIZJ1NP, pCIZJ1P, and pCIZJ1L) had been previously generated by Hu et al. [6]. And the full-length cDNA of the pNDV/AI4 was constructed by Hu et al., previously [14]. Strain JS-14-12-Ch (F gene: KJ184584, HN gene: KJ184603) was isolated from a vaccinated broiler flocks. The virus was plaque purified on chicken embryo fibroblast (CEF) cultures for three times [9]. Sequence analysis of the HN gene indicates that JS-14-12-Ch possesses amino acid changes (E347K and G362A) in and around the linear epitope of HN gene.

### 2.3. Phylogenetic Analysis

Nucleotide sequence editing, analysis and prediction of amino acid sequences for the F and HN protein and alignments were conducted using the Clustal W multiple alignment method in the MegAlign 7.1.0 program of the Lasergene package (DNASTAR Inc., Madison, WI, USA). Phylogenetic trees based on the *F* and *HN* gene were both constructed by the program MEGA5 (Version 5.2), using the neighbor-joining method algorithm and the accession numbers of these NDVs were shown in the phylogenetic tree.

### 2.4. Replacement of Hemagglutinin–Neuraminidase Gene of the Recombinant Virus NDV O/AI4 and Virus Rescue

The infections of the variant sub-genotype VIId NDV strains with E347K mutation on the HN protein occurred frequently in China. To decrease the antigenic difference between NDV/AI4 and the predominant strains, HN gene from JS-14-12-Ch was used to replace the corresponding region in the full-length cDNA of pNDV/AI4. Briefly, the 1561 bp Sac II-Spe I fragment (nucleotides 6540–8101) containing the open reading frame (ORF) of HN gene of NDV JS14-12-Ch was amplified with primers NDVHNP1 (5’-CCGCGGCTGCCCTGGC-3’) and NDVHNP2 (5’-GATGTCGTCTTCCCAACC ATCCTAT-3’). The PCR product was then digested with Sac II and Spe I restriction enzymes and the 1561 bp fragment was then introduced back to the same site in the genomic cDNA clone pNDV/AI4 for constructing the plasmid pO/AI4. Recovery of the recombinant NDV using pO/AI4 was performed as described previously [14,15,16], and the rescued virus was designated as NDV O/AI4. The biologic characterization of the rescued viruses was evaluated by standard determined the mean death time (MDT), the intracerebral pathogenicity index (ICPI), and the intravenous pathogenicity index (IVPI) [19].

### 2.5. Cross Hemagglutination Inhibition Test and Virus Neutralization Test

To measure the antigenic difference between the three vaccine strains (LaSota, NDV/AI4 and NDV O/AI4) and the isolated variant strain JS-14-12-Ch, the cross hemagglutination inhibition(HI) and virus neutralization test was performed as described by Cho and Li [2,20]. The anti-serum against LaSota, NDV/AI4, NDV O/AI4 and JS-14-12-Ch were prepared from the specific-pathogen-free (SPF) chickens vaccinated with inactivated oil-emulsion LaSota, NDV/AI4, NDV O/AI4 and JS-14-12-Ch, respectively. Three weeks after vaccination, serum was collected and stored at −70 °C until use. The antigenic relatedness between vaccine strains were expressed in R value, as described by Archetti and Horsfall [21]. The following formula was used: *R* = $\sqrt{r1*r2}$, where *r*1 is the titer of strain A with antiserum B divided by the titer of strain A with antiserum A and *r*2 is the titer of strain B with antiserum A divided by the titer of strain B with antiserum B. A result of 0.67 ≤ *R* ≤ 1.5 indicates no significant antigenic difference between the two viruses, whereas 0.5 ≤ *R* ≤ 0.67 indicates a minor difference between the two viruses. An *R* value of *R* < 0.5 indicates a major difference between the two virus strains [22].

### 2.6. Preparation of Vaccines

The inactivated oil emulsion vaccines were prepared as Hu et al. described previously [14]. NDV O/AI4, NDV/AI4 and LaSota were cultured in embryonated chicken eggs and the infective allantoic fluids were collected for each vaccine virus. Viruses were inactivated by treatment with 0.7% formaldehyde at a proportion of 43:7 at 4 °C for 72 h. Inactivated viruses were mixed with Tween-80 (polysorbate, Tianjin Chemical Experiment Plant, Tianjin, China) at a ratio of 100:4 at 37 °C, which constituted the aqueous phase. The oil phase was composed of 100 parts white oil (Hangzhou Petrochemical Company, Hangzhou, China) and four parts Span 80 (sorbitan monooleate, Tianjin Chemical Experiment Plant, Tianjin, China). One volume of the aqueous phase was emulsified in three volumes of the oil phase to obtain oil-emulsion vaccines.

### 2.7. Experimental Design

The birds were housed in isolators under negative pressure with food and water provided ad libitum. To determine an appropriate immunization volume of different inactivated oil emulsions, smaller pilot studies with vary doses of three vaccines had been proceeded before. Three groups of twenty SPF chickens were vaccinated through subcutaneous route. Group 1 was inoculated with 40 μL of inactivated oil emulsion LaSota, group 2 was inoculated with 15 μL of inactivated oil emulsion NDV/AI4 and group 3 was inoculated with 15 μL of inactivated oil emulsion NDV O/AI4, respectively. The negative control group consisted of five chickens which received PBS via the same route. Three weeks after vaccination, the antibodies of all birds were tittered and the six vaccination groups were further grouped by antibody titers: group LaSota with 6log_2_, AI4 with 6log_2_, and O/AI4 with 6log_2_, respectively.

All birds were challenged intraocularly and intranasally with 10^6^ 50% egg lethal dose (EID_50_) in a volume of 0.1 mL of the variant sub-genotype VIId NDV strain, JS-14-12-Ch. All birds were also observed daily for clinical signs of disease until the end of the experiment, two weeks post challenge. Tracheal and cloacal swabs were taken at three, five, and seven days post challenge (dpc) for virus isolation and titration. To evaluate the replication of JS-14-12-Ch in chicken tissues at 3 dpc, viral load in visceral organs was titrated (lgTCID_50_/0.1 g tissue) in CEF cells by the method of Reed and Muench and the collected tissues were also fixed by immersion in 10% neutral buffered formalin. All samples were embedded into paraffin, and sectioned at 3 μm and then routinely deparaffinized and stained with hematoxylin and eosin for histopathological examination.

### 2.8. Statistical Analysis

Frequencies of virus isolation were analyzed for significance by Fisher’s exact test. Group mean titers of virus from oral swabs were analyzed by the independent samples *t* test.

## 3. Results

### 3.1. Phylogenetic Analysis

**Figure 1:**
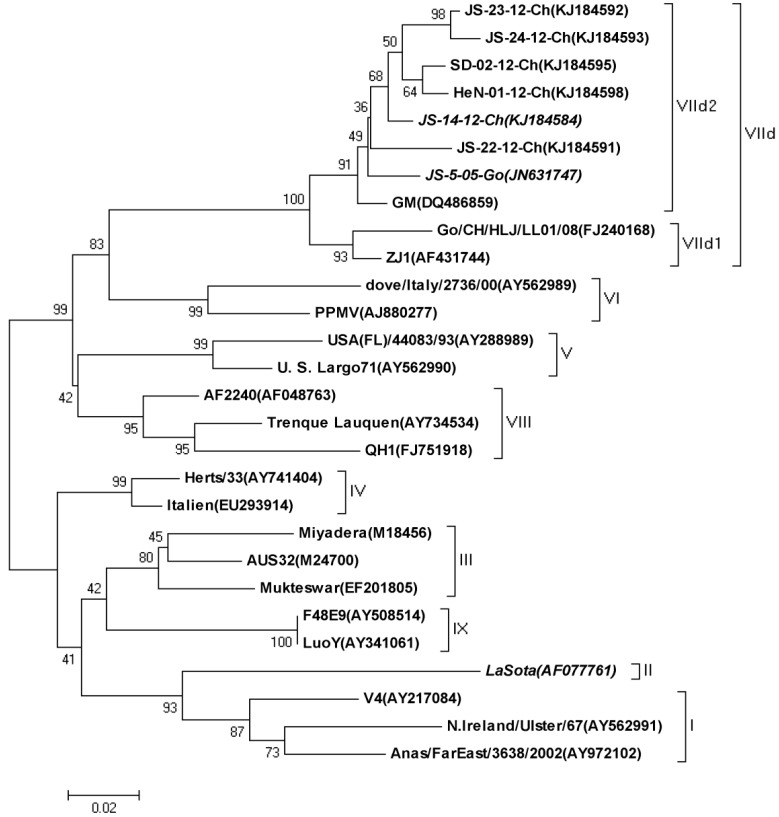
Phylogenetic analysis of the three Newcastle disease virus (NDV) strains based on the variable region of the F gene (nucleotides, 47-420).

Phylogenetic analysis of three NDV strains characterized in this paper and 25 reference NDV strains from GenBank was performed based on the sequences of the variable region of the F (Fusion) genes (nucleotides 47 to 420) (Figure 1). The amino acid similarity between LaSota and the circulating virus JS-14-12-Ch was 83.6% for the F protein and 81.3% for the HN protein.

The similarity between NDV/AI4 and JS-14-12-Ch was 99.2% for the F protein and 97.6% for the HN protein. This comparison confirms that genetic distance between LaSota and the circulating strain is greater than that between the circulating strains. The two sub-genotype VIId NDV strains were close in the phylogenetic tree and some amino acid alterations were detected in and around the linear epitope of the HN protein which contained the E347K and G362A co-mutation.

### 3.2. Recovery of the Recombinant NDV O/AI4

In an effort to further investigate the role of the HN protein in NDV shedding and transmission, we replaced the HN gene of strain NDV/AI4 with that of JS-14-12-Ch by reverse genetics. The supernatant of the transfected cells was injected into the allantoic cavity of nine- to 10-day-old SPF chicken embryos. The recovered chimeric virus NDV O/AI4 was serially passed 10 times in nine-day-old SPF chicken embryos and the HA titer for each passage was detected as 2^10^. By sequencing the HN protein of each passage, none of the nucleic acids of the HN protein mutated. All three viruses were titrated before inactivation (NDV/AI4 = 10^8.7^ EID_50_/0.1 mL, LaSota = 10^9.3^ EID_50_/0.1 mL and NDV O/AI4 = 10^8.5^ EID_50_/0.1 mL, respectively).

### 3.3. Cross Hemagglutination Inhibition (HI) Test and Antigenic Relatedness Analyse

To evaluate the antigenic diversity of different strains, the cross HI assays were performed to calculate the *R* value. The *R* values between JS-14-12-Ch and the two vaccine strains, LaSota and NDV/AI4, were less than 0.5, indicating that an obvious significant antigenic difference existed between the vaccine strains and the variant NDV strain circulating in China. Meanwhile, no significant antigenic difference was observed between JS-14-12-Ch and NDV O/AI4, as evidenced by the *R* value being equal to 1.0. In addition, the antigenic difference was further confirmed by the virus neutralization test (Table 1). An obvious antigenic variation between the genotype VII NDV strain and the commercial vaccine strain LaSota was observed, which was consistent with the previous research [23]. However, antigenic variations have been also found between AI4 and the circulating strain JS-14-12-Ch, indicating an evolution within the sub-genotype VII.

### 3.4. Virus Shedding

Initially, the clinical signs of viral infection were observed by three dpc, as most birds in the control group were depressed, had diarrhea, and consumed less food and water. Four of 12 LaSota-vaccinated birds developed such severe clinical signs while all birds vaccinated with the other two genotype VII vaccines (NDV/AI4 and NDV O/AI4) appeared normal without any clinical symptoms. All birds of the control group died within five days post-challenge and the rest survived at the end of animal experiment.

**Figure 2:**
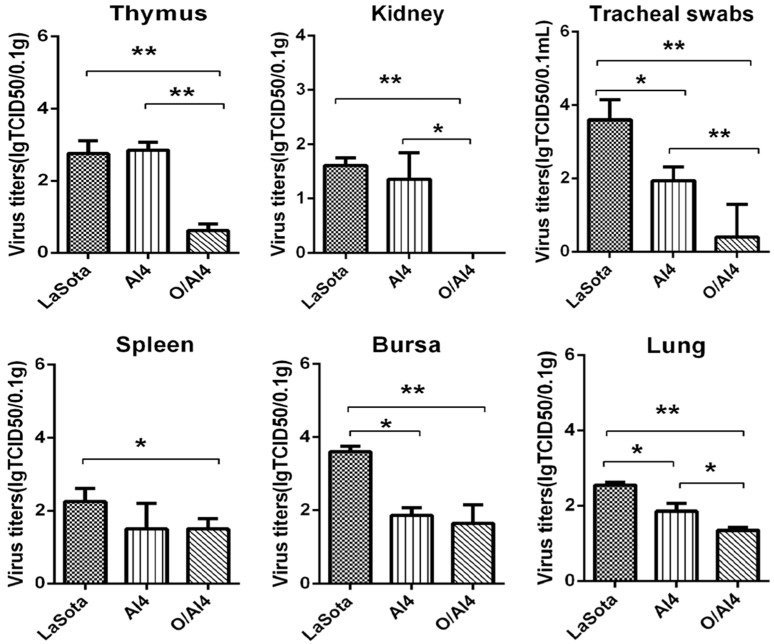
Viral load in visceral organs and tracheal swabs of chickens infected with JS-14-12-Ch at three days post challenge. Virus titers of the collected tissues were assessed in CEF cells and are presented as lgTCID_50_/0.1 g tissue and Virus titers of the collected tracheal swabs were assessed in CEF cells and are presented as lgTCID_50_/0.1 mL. * *p* < 0.05; ** *p* < 0.01.

Among the vaccinated groups, there was a significant decrease in the number of birds shedding the virus from the trachea and cloaca at three, five and seven dpc in group O/AI4 than in the other two groups (Table 2), whereas the geometric mean virus titer of the O/AI4 group was significantly lower when compared with that of the LaSota or AI4 (*p* < 0.01) groups at three dpc in trachea swabs (Figure 2). Moreover, the viral loads in the kidney, thymus and lung of the group O/AI4 were significantly decreased when compared with that of the other two groups. Furthermore, the viral load in the bursa of the LaSota group was significant higher than that of the other two groups and the viral load in the spleen of the LaSota group was only significantly higher than that of group O/AI4.

### 3.5. Histopathology

**Figure 3:**
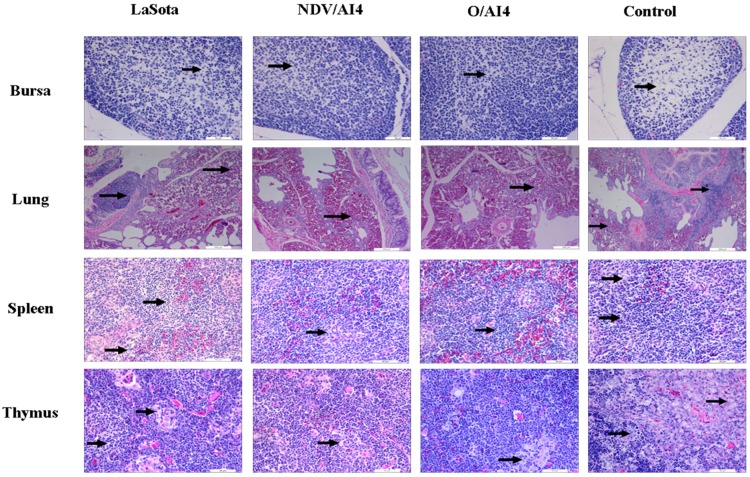
Photomicrographs illustrating hematoxylin and eosin (HE) staining on sections of the bursa of the Fabricius, lung, spleen and thymus at 3 dpc. Magnification for panels lung, *n* = 100×, bars = 200 μm; all other panels, 400×, bar = 50 μm. First row: bursa tissue samples from LaSota, NDV/AI4, O/AI4 vaccinated groups showed moderate edema (black arrows), while tissue from the control group showed severe edema. Second to fourth row: spleen, thymus and lung tissue samples from LaSota vaccinated group showed mild lymphocytosis (black arrows) which was more severe than that from the NDV/AI4, O/AI4 vaccinated groups. Moderate lymphocytosis along with lymphoid necrosis was observed in the tissues from the control group.

The results of the histopathological evaluation are presented in Table 3 and Figure 3. All three vaccinated groups displayed very similar histological changes in multiple organs and tissues at 3 dpc, and the most extensive damage was present in the lymphoid organs. Mild to moderate lymphocytosis along with lymphoid necrosis was observed in the spleen, thymus and lung, while moderate to severe edema was found in the bursa. The LaSota group showed more severe histological changes than the two genotype VII vaccine groups, while the histological changes of the two genotype VII vaccine groups could not be differentiated.

## 4. Discussion

ND vaccination is an important measure to prevent virulent NDV infection and ND vaccines have been widely and frequently used in the Chinese poultry industries [7,9,14,24,25]. However, virulent NDV could be still isolated from ND-vaccinated poultry flocks, which raised the concern of whether the current vaccines are still effective, not only for the protection from clinical disease but also for the reduction of virulent virus shedding and transmission. Currently, the widely used ND vaccine strain LaSota, isolated 70 years ago, belongs to genotype II, while the genotype dominantly prevalent in China is characterized as VIId [10,26,27]. The genetic difference between the two genotypes is considered as the major factor contributing to the vaccination failure in poultry flocks in China [23]. Therefore, there is an urgent need to investigate the molecular mechanisms that cause antigenicity differences and a necessity to develop new matched vaccines.

The HN protein of NDV plays an important role in inducing immune protection against virus infection, and is therefore susceptible to immune pressure to generate antigenic variation more easily [20]. Recently, the variant sub-genotype VIId NDV, with E347K and G362A mutations in the HN protein, has been predominantly circulating in China [27,28,29]. These two mutations are located in and around the HN linear epitope and might further decrease the immune efficacy of the routine vaccine strain against the prevalent genotype VIId viruses [8]. To verify this hypothesis, in this study, we replaced the HN gene of strain NDV/AI4 with that of JS-14-12-Ch by reverse genetics and recovered the recombinant NDV O/AI4. The antigenic variation between the three vaccine strains (NDV O/AI4, NDV/AI4 and LaSota) and the predominant virulent NDV JS-14-12-Ch was analyzed based on the result of in vivo and in vitro experiments. The results indicated that the vaccine strain LaSota showed significant antigenic differences to the variant genotype VIId NDV JS-14-12-Ch, as shown in the result of cross HI and virus neutralization tests. Notably, a significant antigenic difference was also observed between the two sub-genotype VIId strains JS-14-12-Ch and NDV/AI4, while the antigenic difference was not detected between the JS-14-12-Ch and NDV O/AI4 strains which only possessed different a HN gene compared with NDV/AI4. Further analysis showed that the HN protein of NDV/AI4 and NDV O/AI4 shared higher identities in the amino acid sequence, and except for the variation in the linear epitope, the other five epitopes were all conserved (data not shown). Therefore, the mutations E347K and G362A in the HN protein likely resulted in a significant change in NDV antigenicity. 

To further evaluate the influence of the antigenic difference on NDV shedding and transmission, three oil emulsion vaccines, developed based on NDV O/AI4, NDV/AI4 and LaSota, were used to compare their immune-protective efficacy against the prevalent NDVs. Previously, Hu et al. compared the immune efficiency of attenuated sub-genotype VIId NDV/AI4 with the vaccine strain LaSota, and found that NDV/AI4 could induce a higher level of antibodies and therefore provided better protective efficacy [6,14]. In this study, to eliminate the influence of different antibody levels, the vaccination and challenge experiments were performed with the same HI titer of 6log_2_ post-vaccination. As shown in the results, LaSota could not completely protect the vaccinated birds from clinical disease as compared to the other two vaccines. NDV O/AI4 provided protection for more birds in terms of virus shedding, and significantly reduced virus shedding in tracheal swabs compared to the LaSota and NDV/AI4 vaccines. Similarly, NDV O/AI4 could significantly reduce the viral load in the visceral organs which was consistent with the results from the histopathological examination where the O/AI4 group showed only mild histological changes post-challenge. Taken together, our data indicated that the antigenic variation in HN could significant decrease the immune efficacy of the LaSota vaccine strain, and the lower antibody level induced by LaSota could not prevent the vaccinated birds both from the variant virulent NDV infection and clinical signs. Furthermore, even with the same genotype, the NDV O/AI4-based inactivated vaccine could provide better immune potency against the variant genotype VIId NDVs than the NDV/AI4-based vaccine. This result was in agreement with the cross HI and virus neutralization tests, suggesting that the HN antigenic difference was critical in the immune potency of the vaccines. In summary, the vaccines currently used to prevent ND were derived from strains isolated several decades ago. However, there has been a significant gap in the antigenicity between the prevalent NDVs and the vaccine strains. The results reported here indicate that the antigenic similarity between the vaccine strain and the prevalent strain of NDV is important in reducing the shedding of virulent virus, in which the NDV HN protein may play a critical role.

## Figures and Tables

**Table 1:** Coefficients of antigenic similarity (*R*) between the three vaccines and JS-14-12-Ch.

Strains	*R* Value ^c^	345–353 Residues of HN
NDV/AI4	0.35 ^a^, 0.35 ^b^	PDKQDYQIR
O/AI4	0.7 ^a^, 1 ^b^	PDKQDYQIR
LaSota	0.06 ^a^, 0.12 ^b^	PDEQDYQIR

^a^ Chicken embryo cross-neutralization test; ^b^ Cross-hemagglutination inhibition test; ^c^ 0.67 ≤ *R* ≤ 1.5, indicates no significant antigenic difference between the two viruses; 0.5 ≤ *R* ≤ 0.67 indicates a minor difference between the two viruses. *R* < 0.5 indicates a major difference between the two virus strains.

**Table 2:** Frequency of isolation of challenge virus in different vaccine groups.

Group	Vaccine	Antibody Titers (log_2_)	No. Swabs Positive in Virus Isolation/Total No. of at Days Post Challenge					
			3 dpc		5 dpc		7 dpc	
			T ^a^	C ^b^	T	C	T	C
LaSota	LaSota	6	10/12	4/12	4/9	5/9	0/9	3/9
AI4	NDV/AI4	6	9/12	1/12	3/9	2/9	0/9	0/9
O/AI4	NDV O/AI4	6	4/12	0/12	2/9	1/9	0/9	0/9
Control	PBS	0	5/5	5/5	- ^c^	-	-	-

^a^ Tracheal swabs; ^b^ Cloacal swabs; ^c^^Ple^ All birds in the control group died before 5 dpc.

**Table 3:** Distribution and intensity of histological lesions.

Organ	Group			
	LaSota	AI4	O/AI4	Control
Bursa	++	+	+	+++
Lung	++	+	+	+++
Spleen	++	+	+	+++
Thymus	++	+	+	+++

Lesions in bursa were graded on the degree of edema. Lesions in lung, spleen and thymus were graded on degree of lymphocytosis: +, mild lesions; ++, moderate lesions; +++, marked lesions.
